# An easy-to-use CRISPRi plasmid tool for inducible knockdown in *E. coli*

**DOI:** 10.1016/j.btre.2021.e00680

**Published:** 2021-10-09

**Authors:** Robert W. Bradley

**Affiliations:** Department of Life Sciences, Imperial College London, SW2 5LU, United Kingdom

**Keywords:** CRISPRi, Inducible repression, One-pot assembly

## Abstract

CRISPR-interference (CRISPRi) is a powerful genetic tool with wide application in bacteria. In this work a single plasmid system is presented which expresses deactivated Cas9 and a short guide RNA sequence from separate inducible promoters, enabling up to 40-fold repression of target genes with minimal leaky repression in the uninduced state. The plasmid is designed for rapid spacer insertion with easy screening for correct clones, and a simple one-day protocol with >99.9% assembly efficiency is described. This plasmid is made available to the research community with a view to facilitating wider use of CRISPRi in bacterial systems.

## Highlights

• An easy-to-use plasmid tool for CRISPRi transcription repression in bacteria.
• Dual inducible promoters for tight control of CRISPRi components.
• One-day cloning protocol to insert targeting spacer sequences.
• Strong repression of plasmid-borne and chromosomal targets.

## 1 Introduction

In the years since its inception [1] bacterial CRISPR-interference (CRISPRi) has been used for applications ranging from functional screens [2] to synthetic gene circuit design [3]. The mechanism of transcriptional repression and details of guide RNA design have been described in detail elsewhere [1,[4], [5], [6]]. Briefly, the deactivated form of the Cas9 protein (dCas9) lacks nuclease activity but retains the ability for strong RNA-guided DNA-binding which can block RNA polymerase promoter binding or its processivity in transcript elongation. The minimum requirements for the CRISPRi system are the dCas9 protein and a short-guide-RNA (sgRNA) which is a synthetic polynucleotide combining the ‘spacer’ RNA sequence and the trans-activating-CRISPR-RNA (tracrRNA). The 20-nucleotide spacer sequence is complementary to the target DNA sequence, whilst the tracrRNA section acts as a scaffold for RNA-dCas9 binding. The target sequence must be upstream and adjacent to a ‘protospacer adjacent motif’ sequence, 5′-NGG-3′ in the case of the classical *Streptococcus pyogenes* Cas9 system.

The motivation for this work was to build a highly accessible CRISPRi system by combining desirable design elements from previously described plasmids. Such a system would: be based on a single plasmid [4]; have separate inducible control over dCas9 and sgRNA expression [3]; require only the spacer sequence to be inserted [7]; and use a one-pot assembly method with a marker to screen for spacer insertion[8,9]. The design of the resulting plasmid pdCas9-sgRNA-RFP and tests against plasmid-borne and genomic targets are described here.

## 2 Results

### 2.1 Plasmid design

**Fig. 1:**
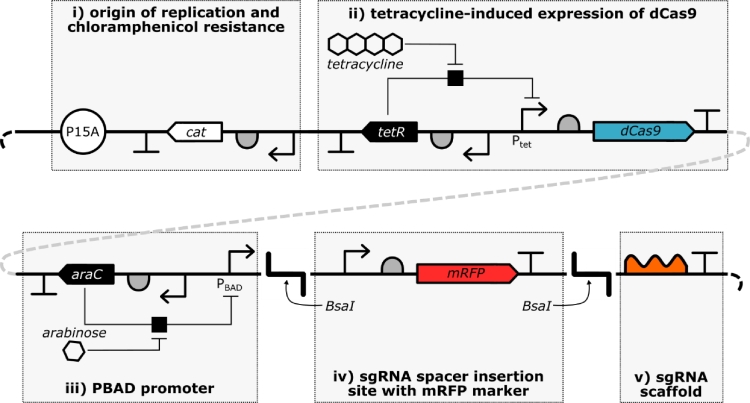
– Plasmid diagram. The inducible knockdown plasmid pdCas9-sgRNA-RFP with highlighted functional modules. Spacer sequences are cloned in place of the mRFP marker cassette (box iv) between BsaI sites.

The plasmid pdCas9-sgRNA-RFP is based on the pCas9-CR4 backbone [10] with inactivation of Cas9 and introduction of an arabinose-responsive promoter and sgRNA scaffold. The key features of this plasmid are the dual inducible expression modules and spacer insertion site with mRFP marker and flanking Type IIS restriction enzyme sites (Fig. 1**,** boxes ii, iii and iv). These enable tight control of knockdown components by separately controlling the induction of dCas9 expression with anhydrotetracycline and the induction of sgRNA expression with arabinose, plus allowing for easy assembly and screening for spacer insertion into the sgRNA scaffold.

### 2.2 One-pot single-day spacer cloning

Spacer sequences are cloned as annealed oligonucleotides into the sgRNA scaffold using a one-pot assembly reaction with the Type IIS restriction enzyme BsaI. The assembly reaction removes the mRFP marker cassette from the plasmid backbone, so that successful clones can be identified through an absence of red fluorescence in the colony. The high-expression mRFP marker cassette allows for identification of colonies by eye on a plate, or it could be used for fluorescence-activated cell sorting in high-throughput applications. Plasmids can then be verified by sequencing before use in the experimental strain, or for the fastest protocol the assembly mix can transformed directly into the experimental strain and correct clones identified through a combination of fluorescence loss and colony PCR with a primer targeting the inserted spacer sequence. Spacer integration efficiency was assessed using 25- and 35-cycle assembly reactions and was high for both conditions (Table 1); using more cycles increases the proportion of completed assemblies, but the 99.9% assembly rate at 25 cycles should be sufficient for most applications. All 15 sequenced non-fluorescent clones contained an inserted spacer; one was found to contain a mutation.

**Table 1:** – One-pot spacer integration efficiency.

Number of cycles a	25	35
**Proportion of fluorescent colonies (unmodified plasmid)**^b^	< 0.1% (7 / > 10,000)	< 0.01% (0 / > 10,000)
**Proportion correct sequence**^c^	93% (14 / 15)	

a During one-pot assembly: one cycle is five minutes at 37 °C and five minutes at 16 °C.

b Proportion of fluorescent colonies aggregated from three independent assemblies. Number of fluorescent colonies shown in brackets.

c Proportion of fifteen non-fluorescent colonies picked for sequencing, aggregated from reactions using nine different spacer sequences. Incorrect clone had mutated spacer inserted.

### 2.3 Strong inducible knockdown of plasmid-based and chromosomal gene targets

**Fig. 2:**
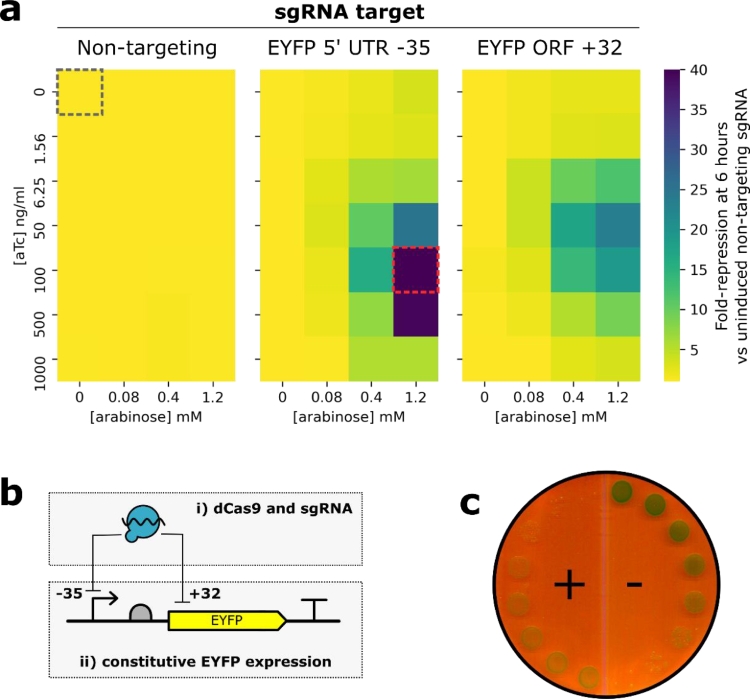
– Inducible knockdown of target genes. (**a**) Heatmaps showing fold-repression of EYFP expression. Each datapoint is the average of three biological replicates, blank-corrected against cells grown without the EYFP expression plasmid. Fold repression is calculated versus the uninduced non-targeting sgRNA sample, highlighted with a black dashed edge. No detectable fluorescence was observed in the sample highlighted with a red dashed edge. (**b**) The EYFP knockdown experiment uses a two-plasmid system: expression of dCas9 and sgRNA from pdCas9-sgRNA-RFP (box i) and constitutive expression of EYFP (box ii). (**c**) Knockdown of chromosomal *gusA*. Dilution series of cells containing pdCas9-sgRNA-gusA+54 were grown on solid media containing X-Gluc substrate; (+) and (-) markers indicate the presence or absence of arabinose and anhydrotetracycline inducers.

The efficacy of dCas9 knockdown was tested against EYFP expression from a plasmid vector. Two spacer sequences were chosen, one targeting the -35 region of the strong constitutive promoter (BBa_J23101) driving EYFP transcription, the other targeting the non-template strand [1] of the EYFP ORF at position 32. A non-targeting spacer was included as a negative control. Quantification of EYFP knockdown and a schematic of the sgRNA targets are shown in Fig. 2a and 2b. Knockdown was strongest with 1.2 mM arabinose and 50–500 ng/ml anhydrotetracycline (aTc): using the spacer targeting the -35 region of the EYFP promoter no EYFP fluorescence was detected indicating extremely strong repression was achieved. The highest aTc concentration was not optimal for repression and there was a significant growth impairment at concentrations > 100 ng/ml (data not shown), possibly due to the toxic effects of dCas9 over-expression [11]. The 6-hour time point was chosen as the average OD_600_ value was ≤1.0 and cells were actively growing; higher fold-repression was observed at later time points (data not shown) but cells are likely becoming starved and stressed. Minimal repression from the non-targeting spacer indicates that knockdown is specific to dCas9 action and not an effect of expression burden. It should be noted that some proportion of the apparent repression of this plasmid-based target may be due to bound dCas9 interfering with plasmid replication and reducing EYFP copy number [12].

Knockdown of a chromosomal gene was also demonstrated by targeting *gusA* (*uidA*), the gene encoding *β*-glucuronidase. A spacer targeting the *gusA* ORF at +54 was cloned into pdCas9-sgRNA-RFP and cells containing the construct were plated on media containing the X-Gluc reporter substrate for *β*-glucuronidase which forms blue precipitate upon hydrolysis. Cells grown in the presence of arabinose and anhydrotetracycline showed a marked reduction in *β*-glucuronidase activity compared to uninduced cells (Fig. 2C).

## 3 Discussion

The CRISPRi plasmid described here allows researchers to rapidly investigate the effects of targeted gene knockdowns in *E. coli* and may also be functional in other Enterobacteriaceae which can host the P15A origin of replication, including species of *Klebsiella* and *Salmonella*. The highly efficient spacer integration method could be used to construct knockdown libraries via a single assembly reaction, making it suitable for massively parallel screening of transformants for phenotypes. The dual-inducible promoter system minimises the plasmid's burden on uninduced cells, whilst allowing for tuneable repression of targets – an important capability for the investigation of essential genes. Plasmid pdCas9-sgRNA-RFP is available from the Addgene repository (#166,005).

## 4 Materials and methods

### 4.1 Strains, culture conditions

Top10 *E. coli* were used for both cloning and knockdown experiments, and were cultured at 37 °C in LB-Lennox medium with antibiotics added at the following concentrations where appropriate: chloramphenicol 25 µg/ml, ampicillin 100 µg/ml.

### 4.2 Cloning

Plasmid pdCas9-sgRNA-RFP was constructed using Gibson assembly and the full annotated sequence is available from the Addgene repository (plasmid #166,005). Briefly, the pCas9-CR4 plasmid (a gift from Kristala Prather, Addgene plasmid # 62,655) was modified to introduce inactivating D10A and H40D mutations to the Cas9 ORF, and other parts were introduced between the 3′ end of the Cas9 ORF and its transcription terminator BBa_B0015 (BioBrick part reference). The araC regulator and P_BAD_ promoter are nucleotides 1:1190 of BioBrick part BBa_I0500 and the mRFP marker is BioBrick part BBa_J04450.

The EYFP expression construct was created using BioBrick assembly and contains the parts BBa_J23101 [strong minimal constitutive promoter] – TAGTGGAGGTTA [strong RBS] – BBa_E0030 [EYFP] – TTAATTAATTAAGGGGACCCTAGAGGTCCCCTTTTTTTATTTT [terminator] in a pUC19 backbone. BioBrick part sequences are available at http://parts.igem.org.

All enzymes were obtained from Thermofisher; restriction enzymes are all FastDigest. Spacer oligos require a 5′ CCAT or AAAC addition to the forward and reverse oligos respectively to create overhangs for annealing into BsaI sites. Spacer oligonucleotide sequences used in this study (5′−3′): non-target-fwd: ccatAACCGTCTAGGCCTAGACTC; non-target-rev: aaacGAGTCTAGGCCTAGACGGTT; EYFP-35-fwd: ccatTTTACAGCTAGCTCAGTCCT; EYFP-35-rev: aaacAGGACTGAGCTAGCTGTAAA; EYFP+32-fwd: ccatGACCAGGATGGGCACCACCC; EYFP+32-rev: aaacGGGTGGTGCCCATCCTGGTC; gusA+54-fwd: ccatGTGGGCATTCAGTCTGGATC; gusA+54-rev: aaacGATCCAGACTGAATGCCCAC.

### 4.3 One-pot spacer assembly

100 fmol annealed phosphorylated spacer oligos were used in a 20 µl reaction with 10 fmol pdCas9-sgRNA-RFP plasmid, T4 DNA ligase buffer, 1 µl T4 DNA ligase and 1 µl BsaI (Eco31I). Temperature cycling: 20 min @ 37 °C; 25 or 35 repeats of (5 min @ 37 °C, 5 min @ 16 °C); hold @ 10 °C. The assembly mix was used to transform chemically competent cells at a 1:10 volumetric ratio.

### 4.4 Fluorescence detection

Overnight cultures of *E. coli* strains were sub-cultured and grown to an OD_600_ of 0.4–0.6, washed once in fresh medium, and resuspended to an OD_600_ of 0.01. Inducers were diluted in LB to 20X stocks, and cells and inducers were combined to a final volume of 200 µl in a Greiner black 96-well plates. Fluorescence (Ex 485 nm/Em 520 nm) and absorbance (600 nm) were monitored using a BMG Labtech Fluostar plate reader at 37 °C with 200 rpm shaking between measurements.

### 4.5 GusA knockdown

10-fold serial dilutions of *E. coli* harbouring pdCas9 with *gusA* targeting spacer were spotted onto plates containing 40 µg/ml X-gluc, plus 1.2 mM arabinose and 100 ng/ml anhydrotetracycline where required. The image of the plate was captured using an Epson Perfection V37 scanner. Brightness and saturation of the image were increased using Inkscape to improve contrast between colonies and the plate.

## Declaration of Competing Interest

There are no conflicts of interest to declare.
